# UNPARTNERED AND CHILDLESS OLDER ADULTS' RISK OF LONELINESS DURING COVID-19 BY COUNTRY CONTEXT

**DOI:** 10.1093/geroni/igac059.1501

**Published:** 2022-12-20

**Authors:** Christine Mair, Bruno Arpino, Nekehia Quashie, Radoslaw Antczak

**Affiliations:** University of Maryland, Baltimore County (UMBC), Baltimore, Maryland, United States; Department of Statistics, Computer Science, Applications, Firenze, Toscana, Italy; University of Rhode Island, Kingston, Rhode Island, United States; Institute of Statistics and Demography, Warszawa, Warminsko-Mazurskie, Poland

## Abstract

Older adults with non-traditional family structures (unpartnered and childless) may be at higher risk for loneliness. Yet, experiences of loneliness during COVID-19 can vary depending on a country’s context (e.g., culture, demography, COVID-19 mitigation policies, severity of the pandemic). We explore associations between older Europeans’ family structure and loneliness to assess risks for those with non-traditional family structures (e.g., unpartnered vs partnered, childless vs parents) during the pandemic across a variety of country contexts. We analyze data from the Survey of Health, Ageing and Retirement in Europe (SHARE) collected before (2019/2020) and twice during (2020, 2021) the pandemic to examine if unpartnered and childless older adults are at higher risk of loneliness, and compare results by multiple indicators of country context (fertility and partnership rates, stringency of COVID mitigation policies, population age composition, and COVID fatality rates). Results of this study can potentially inform current and future pandemic mitigation strategies.

